# Dietary magnesium intake, serum high sensitivity C-reactive protein and the risk of incident knee osteoarthritis leading to hospitalization—A cohort study of 4,953 Finns

**DOI:** 10.1371/journal.pone.0214064

**Published:** 2019-03-25

**Authors:** Sanna Konstari, Laura Sares-Jäske, Markku Heliövaara, Harri Rissanen, Paul Knekt, Jari Arokoski, Jouko Sundvall, Jaro Karppinen

**Affiliations:** 1 Medical Research Center Oulu, Department of Physical and Rehabilitation Medicine, Oulu University Hospital and University of Oulu, Oulu, Finland; 2 Department of Public Health Solutions, National Institute for Health and Welfare, Helsinki, Finland; 3 Department of Physical and Rehabilitation Medicine, Helsinki University Hospital and University of Helsinki, Helsinki, Finland; 4 Finnish Institute of Occupational Health, Oulu, Finland; Medizinische Universitat Innsbruck, AUSTRIA

## Abstract

**Objectives:**

To study whether low dietary magnesium (Mg) intake and serum high sensitivity C-reactive protein (hs-CRP) predict the development of clinical knee osteoarthritis (OA).

**Methods:**

The cohort consisted of 4,953 participants of a national health examination survey who were free of knee and hip OA at baseline. Information on the incidence of knee OA leading to hospitalization was drawn from the National Care Register for Health Care. During the follow-up of 10 years, 123 participants developed incident knee OA. Dietary magnesium intake was assessed on the basis of a food frequency questionnaire from the preceding year. We used Cox’s proportional hazards model to estimate the strength of the association between the tertiles of dietary Mg intake and incident knee OA, adjusted for baseline age, gender, energy intake, BMI, history of physical workload, leisure time physical activity, injuries, knee complaints, the use of Mg supplements, and serum hs-CRP levels.

**Results:**

At baseline, dietary Mg intake was inversely associated with serum hs-CRP even after adjustment for all the potential confounding factors. During the follow-up, the adjusted hazard ratios (with their 95% confidence intervals) for incident knee OA in dietary Mg intake tertiles were 1.00, 1.28 (0.78–2.10), and 1.38 (0.73–2.62); the p value for trend was 0.31. Serum hs-CRP level at baseline did not predict incident knee OA.

**Conclusions:**

The results do not support the hypothesis that low dietary Mg intake contributes to the development of clinical knee OA, although Mg intake is inversely associated with serum hs-CRP level.

## Introduction

Knee osteoarthritis (OA) is a chronic disease that affects the knee joint and is characterized by an imbalance between the degenerative and regenerative processes which lead to overall degeneration and abnormal remodelation of the knee joint. It also affects joint tissues, including cartilage, meniscus, subchondral bone, synovium, ligaments, tendons, and muscles. [1] In Finland, 6.1% of men and 8.0% of women have been clinically diagnosed with knee OA [2]. As knee OA is a major cause of disability in western populations and causes major socioeconomic costs, better understanding of the pathogenesis and risk factors of OA, as well as knowledge of preventive methods, are warranted. The current understanding supports the assumption that knee OA pathogenesis is a combination of biomechanical and biochemical factors. The most widely known predisposing factors for knee OA are age, female gender, high body mass index (BMI), heavy physical workload, and previous knee injuries [1, 3–7]. Still, the pathophysiology and etiology of knee OA remains unclear in many ways.

It has been hypothesized that chronic low-grade inflammation has a role in OA pathogenesis. Previous studies have found elevated serum C-reactive protein (CRP) levels in OA patients in comparison to those among participants without OA [8]. A longitudinal study showed a significant direct relation between serum CRP and both incident OA and OA progression [9]. However, some studies have found no association between serum CRP and prevalent, incident, or progressive knee, hip or hand OA [10]. A recent systematic review on biomarkers for OA also found evidence of prognostic CRP values to still be inconclusive [11].

Magnesium (Mg) is an important micronutrient for human health and is associated with immune responses [12]. Some studies have demonstrated that low levels of dietary Mg intake are associated with increased serum CRP/high sensitivity CRP (hs-CRP) levels [13, 14]. A meta-analysis and systematic review [15] also indicates that dietary Mg intake is inversely associated with serum CRP levels. Among patients with early radiographic knee OA, both dietary and serum Mg were inversely associated with serum hs-CRP levels [16]. Only two cross-sectional studies have examined the associations between dietary Mg intake and knee OA, and both found a modest inverse association between Mg intake and radiographic knee OA [17, 18]. No prospective study has focused on the association between dietary Mg intake and incident knee OA.

We hypothesized that high dietary Mg intake through alleviating low-grade inflammation would provide protection against knee OA. To test this hypothesis, we evaluated the cross-sectional association between Mg intake and hs-CRP and studied their prediction of incident knee OA leading to hospitalization in a cohort representative of the Finnish adult population.

## Materials and methods

### Study population

The present study is based on the Health 2000 Survey, which was carried out in Finland between September 2000 and March 2001 [19]. The nationally representative population sample was formed using a two-stage stratified cluster sampling method of the adult population aged 30 years and over living in mainland Finland. We regionally stratified the sample frame according to the five university hospital regions. In the first stage of sampling, 80 healthcare districts were sampled as a cluster. In the second stage, a random sample of individuals from each of the 80 healthcare districts, altogether 8,028 men and women, was drawn from a nationwide population register. We excluded those who had hip or knee OA at baseline from the study. Written informed consent was obtained from all the participants. The study was approved by the Ethical Committee for Research in Epidemiology and Public Health at the Hospital District of Helsinki and Uusimaa in Finland.

Of the sample, 6,354 participated in the baseline health examination and of these, 5,763 responded to the food frequency questionnaire (FFQ). Four hundred fifty had prevalent knee OA and 247 hip OA diagnosed by a physician during the clinical examination. Of the remaining 5,164 subjects, 211 had missing data in any necessary determinant. After excluding these, the current study cohort consisted of 4,953 participants, who were followed up until December 31, 2010.

### The baseline health examination

The implementation of the Health 2000 Survey, described in detail elsewhere [2, 19, 20], involved a home interview followed by a comprehensive health examination with an interview on joint symptoms, self-administered questionnaires, anthropometric measurements, drawing of fasting blood samples, and a standardized clinical examination by physicians who applied preset diagnostic criteria. The health examinations started on the 11^th^ of September 2000 and ended on the 2^nd^ of March 2001.

In brief, prevalent knee OA was diagnosed if some of the following conditions were fulfilled: 1) previous knee arthroplasty, even in the absence of convincing evidence of the diagnosis of knee OA, 2) typical knee OA symptoms and either of the following (even in the absence of abnormality in physical status): history of previously diagnosed knee OA (even in the absence of documentation); or documented knee OA diagnosis even in the absence of convincing evidence of the diagnosis, or 3) limitation in joint range of motion, motion tenderness and bony deformity suggesting knee OA, but insufficiently indicative history to enable a conclusive diagnosis. Prevalent hip OA was diagnosed if some of the following conditions were fulfilled: 1) previous hip arthroplasty, even in the absence of convincing evidence of the diagnosis of hip OA, 2) typical hip OA symptoms and either of the following (even in the absence of abnormality in physical status): history of previously diagnosed hip OA (even in the absence of documentation); or documented hip OA diagnosis even in the absence of convincing evidence of the diagnosis, or 3) at least slight restrictions in the range of motion, especially if combined with tenderness associated with motion tenderness. A detailed description of the clinical examination and the criteria used are provided elsewhere. [2, 19–21] Knee radiographs were taken of a subsample of participants in the Health 2000 Survey to validate the clinical diagnosis of knee and hip OA for the current study; the agreement between clinical and radiological diagnosis using the Kellgren and Lawrence grading scale was moderate [22].

As described in detail earlier [20, 21], previous injury was defined as self-reported traumatic lower limb injury or an injury of the meniscus or the cruciate ligament of the knee, diagnosed by a clinician. All injuries sustained since the baseline examination were classified by a physician according to the International Classification of Diseases (ICD) 10^th^ revision, on the basis of all the information available in the medical history, the symptoms, and the physical findings of the clinical examination. Musculoskeletal injuries were accounted for only if they had led to permanent damage or to any continuing impairment or complaint. Knee complaint was defined as difficulty in walking or limping due to discomfort or trouble in the knee during the last month. This information was elicited using a standardized interview on musculoskeletal symptoms.

High-sensitivity C-reactive Protein (hs-CRP) was determined from frozen serum samples in 2003 by an immunoturbidimetric test (Orion Diagnostica, Espoo, Finland) using the Optima analyzer (Orion Diagnostica) at the Biochemistry Laboratory, National Institute for Health and Welfare (Helsinki, Finland). The hs-CRP results were classified into three groups as follows 1) < 1.0 mg/L, 2) 1.0–3.0 mg/L, and 3) > 3.0 mg/L according to the ACC/AHA 2014 Guideline [23]. During the course of the measurements, which lasted four months in 2003, the interassay coefficients of variation (CV%) of the CRP determinations were 6.8% (at the mean level of 1.36 mg/L) and 3.6% (at the mean level of 3.10 mg/L) according to 150 control samples analyzed at the beginning and at the end of the daily analysis series. To ensure standardization of measurements, the laboratory took part in External Quality Assessment Schemes organized by Labquality (Helsinki, Finland). The systematic error (bias) (mean ± SD) estimated was -1.3 ± 18.4%.

The home interviews have been previously described in detail [19–21, 24]. During the home interviews we assessed the history of physical workload by eliciting how many years the participants had been exposed in their current or past jobs to heavy physical work involving lifting, carrying or pushing loads over 20 kg for an average of at least 10 times per working day. The information was classified as follows: 1) 0 years, 2) 1–12 years, 3) 13–24 years, or 4) more than 24 years. The information on leisure-time physical activity was elicited via a self-administered questionnaire and categorized as 1) little or irregular leisure-time physical activity and 2) regular fitness training at least three hours per week or regular competitive sports training several times per week. Weight and height were measured in the health examination in light clothing without shoes, and body mass index (BMI, kg/m^2^) was calculated. Participants were classified into four groups according to BMI: 1) < 25.0 kg/m^2^, 2) 25.0–29.9 kg/m^2^, 3) 30.0–34.9 kg/m^2^, and 4) > 35.0 kg/m^2^. [19–21, 24].

### Dietary magnesium intake

Dietary Mg intake was assessed on the basis of a validated self-administered FFQ from the preceding year tailored for this survey [25, 26]. The FFQ had previously been validated in another Finnish survey [27]. The questionnaire listed 128 commonly used food items, mixed dishes, and alcoholic beverages grouped as follows: dairy products, cereal products, fat spreads, vegetables, potatoes, pasta and rice, meat, fish, poultry and eggs, fruit and berries, desserts, sweets and snacks, and beverages. There was also space to add products that were used but not mentioned in the questionnaire. The frequency of the foods used was assessed on a scale of 1 to 9; 1) never or seldom, 2) 1–3 times per month, 3) once per week, 4) 2–4 times per week, 5) 5–6 times per week, 6) once per day, 7) 2–3 times per day, 8) 4–5 times per day, and 9) 6 or more times per day. To assess portion size, example predesigned portion sizes were printed on the questionnaire to which participants were asked to compare their own typical portion sizes.

Average daily Mg intake was calculated using the National Finnish Food Composition Database (Fineli) [28]. Regarding the main dietary sources of Mg, among men, 28% of dietary Mg intake was from cereal, 18% was from dairy, 12% was from non-alcoholic drinks, 12% was from vegetables, and 32% was from other sources with less than 10% contribution each. Among women, 28% of dietary Mg intake was from cereal, 18% was from dairy, 13% was from vegetables, 10% was from non-alcoholic drinks, and 31% was from other sources, with less than 10% contribution each of the total intake [25].

Reliability was evaluated by performing repeated FFQ five to nine months after the baseline FFQ. The FFQ was sent to 198 study participants, of whom 184 returned the questionnaire. The intraclass correlation coefficient for Mg intake was 0.56 among men and 0.60 among women. Among men, the mean dietary Mg intake decreased non-significantly, from 446 mg/day to 427 mg/day (p = 0.18), and among women, significantly, from 418 mg/day to 389 mg/day (p = 0.01). [25] In a validation study, Mg intake based on baseline FFQ also proved satisfactory in comparison to a three-day food record [26].

The FFQ also included questions about the regular use of supplements. The use of magnesium supplements was defined as a record of any single-ingredient magnesium supplement or any combination supplement containing magnesium.

### The follow-up

The follow-up information on the incidence of clinical knee OA was drawn from the National Care Register for Health Care. All Finns have a social security number on the basis of which their health care data are recorded in the register data. All the study participants gave their consent for the use of their register data and therefore this study had no loss to follow-up. The patients treated in a hospital with a diagnosis of knee OA were identified using the International Classification of Diseases, 10^th^ revision (ICD-10); code M17. We decided in advance not to distinguish primary from secondary knee OA on the basis of lacking recommendations for the methods and reproducibility of such differentiation. The follow-up period began at the baseline examination in 2000–2001 and continued until the first hospitalization for knee OA, death, or the end of 2010; whichever came first.

### Statistical analysis

Cross-sectional associations between baseline characteristics, including the known risk factors for OA, hs-CRP and dietary Mg intake, were analyzed using the general linear model. Model-adjusted means were reported in categories of the independent variables [29]. Cox’s proportional hazards model was used to estimate the strength of association between tertiles of dietary Mg intake and the risks of developing knee OA. We constructed two main models: adjusting for age, gender and energy intake; and a full model in which BMI, history of physical workload, leisure time physical activity, injuries, use of Mg supplements, serum hs-CRP level and difficulty in walking due to disorder or complaint in knees were also entered as potential confounding factors. The factors were selected from those available on the basis of prior assumptions of determinants of knee OA and dietary Mg intake, of which age, gender, BMI, history of physical workload and injuries are major risk factors for knee OA [1, 7, 30]. Adjusted hazard ratios (HRs) with 95% confidence intervals (CIs) were estimated in the model’s categories of predictors. Effect modification was studied by entering multiplicative first-degree interaction terms of each variable and dietary Mg intake, one by one, into the full model. We used the SAS System for Windows, version 9.1 (SAS Institute, Inc., Cary, NC) for all the analyses.

## Results

Table 1 shows the distributions of baseline characteristics and their cross-sectional associations with dietary Mg intake. For the whole cohort, the mean intake was 432 mg/day (the lowest value 119, lower quartile 335, median 415, upper quartile 512, the highest value 1352, SD 141 mg/day). For the study population, the mean serum hs-CRP concentration was 2.0 mg/L (the lowest value 0, lower quartile 0.24, median 0.67, upper quartile 1.85, the highest value 191, SD 5.49 mg/L). Mean BMI was 26.6 kg/m^2^ (the lowest value 12.3, lower quartile 23.4, median 26.0, upper quartile 29.2, the highest value 53.8, SD 4.53 kg/m^2^). Dietary Mg intake showed statistically significant associations with age, gender, BMI, leisure time physical activity, serum hs-CRP level, and the use of Mg supplements. When the participants with prevalent OA were included in a corresponding analysis, the Mg intake was, adjusted for all the covariates, among those without and with knee OA 433 mg/day and 439 mg/day (p = 0.09), respectively.

**Table 1:** Baseline characteristics and their cross-sectional associations with magnesium intake.

Factor	Category	Number of subjects	Mg intake (mg/day) adjusted for age, gender and energy intake, mean^a^	SD	Mg intake (mg/day) adjusted for all covariates, mean^a^
Age, years					
	30–54	3309	429	140	428
	55–64	862	445	139	445
	65–74	530	439	150	439
	75–99	252	423	162	424
	p value^b^		< 0.0001		< 0.0001
Gender					
	Male	2263	422	142	422
	Female	2690	441	140	441
	p value^b^		< 0.0001		< 0.0001
BMI, kg/m^2^					
	< 25.0	1954	429	133	428
	25.0–29.9	1987	433	142	433
	30.0–34.9	783	436	145	438
	≥ 35.0	229	442	176	447
	p value^c^		0.001		0.0001
Manual handling of loads of > 20kg, years					
	0	2637	432	136	432
	1–12	900	429	142	430
	13–24	649	438	153	438
	> 24	767	432	144	431
	p value^c^		0.073		0.10
Leisure time physical activity					
	Little or irregular	4035	431	140	431
	Regular	918	438	143	438
	p value^b^		0.011		0.010
Injuries					
	No injury	4780	432	141	431
	Previous injury	173	431	141	438
	p value^b^		0.79		0.58
Hs-CRP, mg/L					
	< 1.0	2973	435	137	436
	1.0–3.0	1214	430	144	429
	> 3.0	766	427	153	422
	p value^c^		0.002		0.0002
Knee complaint					
	No	4427	432	140	432
	Yes	526	436	150	435
	p value^b^		0.24		0.29
Use of Mg supplements					
	No	4326	431	140	431
	Yes	627	438	150	437
	p value^b^		0.033		0.046

^a^Adjusted mean value (general linear model),

^b^*P* value for heterogeneity,

^c^*P* value for trend.

Energy intake, mean 2294 kcal/day, SD ± 797 kcal/day, partial correlation to Mg intake as adjusted for all covariates 0.87.

During the follow-up of 47,348 person-years, 123 new cases of knee OA emerged. The adjusted HRs with 95% CIs for incident knee OA are shown in Table 2. After adjustment for age, gender and energy intake, the HRs (95% CIs) for incident knee OA in tertiles of dietary Mg intake were 1.00, 1.21 (0.74–1.99), and 1.40 (0.74–2.63); p for trend = 0.30. After excluding participants who used Mg supplements and adjusting for all covariates, the corresponding HRs were 1.00, 1.28 (0.78–2.10), and 1.38 (0.73–2.62); p for trend = 0.31. BMI, number of years of handling heavy manual loads and prevalent knee complaints significantly predicted incident knee OA leading to hospitalization.

**Table 2:** Hazard ratios (HRs) with their 95% confidence intervals (CIs) of incident knee osteoarthritis between categories of various factors.

Factor	Category	All	Cases	HR (95% CI), adjusted for age, gender and energy intake	HR (95% CI), adjusted for all covariates
Age					
	30–54	3309	72	1	1
	55–64	862	27	1.49 (0.96–2.32)	1.08 (0.68–1.72)
	65–74	530	18	1.72 (1.03–2.88)	1.27 (0.74–2.17)
	75–99	252	6	1.37 (0.59–3.16)	1.06 (0.45–2.50)
	p value^a^			0.12	0.87
Gender					
	Male	2263	51	1	1
	Female	2690	72	1.20 (0.83–1.72)	1.26 (0.86–1.84)
	p value^a^			0.32	0.23
BMI, kg/m^2^					
	< 25.0	1954	26	1	1
	25.0–29.9	1987	53	1.99 (1.24–3.19)	1.90 (1.17–3.07)
	30.0–34.9	783	32	3.02 (1.79–5.10)	2.84 (1.64–4.90)
	≥ 35.0	229	12	3.80 (1.91–7.55)	3.37 (1.62–7.02)
	p value^b^			< 0.0001	< 0.0001
Manual handling of loads of > 20kg, years					
	0	2637	50	1	1
	1–12	900	21	1.26 (0.76–2.11)	1.18 (0.71–1.97)
	13–24	649	22	1.88 (1.12–2.12)	1.71 (1.03–2.85)
	> 24	767	30	2.04 (1.26–3.31)	1.80 (1.12–2.92)
	p value^b^			0.002	0.007
Leisure time physical activity					
	Little or irregular	4035	98	1	1
	Regular	918	25	1.17 (0.75–1.82)	1.31 (0.84–2.06)
	p value^a^			0.49	0.25
Injury					
	No injury	4780	116	1	1
	Previous injury	173	7	1.76 (0.82–3.78)	1.45 (0.67–3.12)
	p value^a^			0.18	0.37
Hs-CRP, mg/L					
	< 1.0	2973	64	1	1
	1.0–3.0	1214	37	1.35 (0.90–2.03)	1.05 (0.69–1.61)
	> 3.0	766	22	1.31 (0.80–2.13)	0.96 (0.57–1.62)
	p value^a^			0.29	0.95
Knee complaint					
	No	4427	96	1	1
	Yes	526	27	2.33 (1.51–3.58)	2.03 (1.31–3.15)
	p value^a^			0.0004	0.003
Use of Mg supplements					
	No	4326	102	1	1
	Yes	627	21	1.36 (0.85–2.19)	1.46 (0.91–2.36)
	p value^a^			0.22	0.13
Energy intake, kcal/day^c^					
		4953	123	1.13 (0.96–1.34)	1.01 (0.78–1.29)
	p value^b^			0.15	0.96
Magnesium intake, mg/day					
	< 362	1644	33	1	1
	362–474	1653	41	1.21 (0.74–1.99)	1.28 (0.78–2.10)
	> 474	1656	49	1.40 (0.74–2.63)	1.38 (0.73–2.62)
	p value^b^			0.30	0.31

^a^p value for heterogeneity,

^b^p value for trend,

^c^HR per one SD: 797.

Adjustment for only age, gender, and energy intake revealed a slight, statistically non-significant association between higher serum hs-CRP level and incident knee OA (Table 2). This association was attenuated after adjusting for all covariates.

We observed no significant interaction between Mg intake and CRP and other covariates.

## Discussion

Our results are in contrast with the hypothesis that high dietary Mg intake provides protection against knee OA. Moreover, serum hs-CRP did not predict incident clinical knee OA. The inverse association between Mg intake and hs-CRP level that we found was, however, in line with previous studies.

Two cross-sectional studies of Chinese (n = 1,626) [17] and African Americans (n = 665) and Caucasians (n = 1,447) [18] found that low levels of dietary Mg intake increased the risk of knee OA. Both studies used the Food Frequency Questionnaire (FFQ) to assess dietary Mg intake. In the study by Zeng et al., the correlation coefficient between the used semi-quantitative FFQ and the 24-h recall test for measuring Mg intake was 0.53. The study by Qin et al. [18] used the validated FFQ [31]. Knee OA was diagnosed from a knee radiograph using the Kellgren-Lawrence classification. The study by Zeng et al. [17] also assessed radiographic joint space narrowing and osteophytes. In the study by Zeng et al. [17], the multi-variable model was adjusted for age, BMI, gender, educational level, activity level, total energy intake, smoking status, alcohol drinking status, fiber intake, protein intake, zinc intake, calcium intake, iron intake, nutrient supplementation, diabetes and hypertension, whereas in the study by Qin et al. [18], the multi-variable model was adjusted for age, gender, BMI, smoking status, alcohol drinking, education, and total energy intake. The cross-sectional associations found between dietary Mg intake and knee OA may indicate consequences of knee OA rather than any causal chains to its development. No prospective study has previously focused on the association between dietary Mg intake and incident knee OA.

The hypothesis that low dietary Mg intake contributes to the development of knee OA is biologically plausible. The most widely accepted theory of the pathological pathway is that it is through low-grade systemic inflammation, which has been suggested to play a role in OA. Serum hs-CRP is considered the most sensitive biomarker for low-grade systemic inflammation. Elevated serum hs-CRP has predicted cartilage loss associated with OA, as well as poorer outcomes after total knee joint replacement in several studies [32–34]. However, a recent systematic review on biomarkers for OA found that the evidence of a prognostic value of CRP on OA is still inconclusive [11]. There is also evidence that Mg may have direct effects on cartilage tissue by inhibiting chondrocalcinosis on the chondrogenic differentiation of mesenchymal stem cells, and on chondrocyte differentiation and viability at intracellular level. However, the intracellular pathway remains unclear [35].

Some studies, including a meta-analysis and systematic review, indicate that dietary Mg intake is inversely associated with serum CRP/hs-CRP levels [13–15]. Animal studies have reported elevated levels of pro-inflammatory cytokines (such as interleukin-6, tumor necrosis factor alpha), which have been suggested to be potential components of pain, in Mg-deficient animals [36, 37]. In addition, plasma substance P–a known cytokine production stimulator–has been detected in Mg deficiency [38], as has immune cell activation [39]. Even though evidence seems to exist of a relation between Mg deficiency and the inflammatory/immune response, the underlying process for the activation of inflammatory mechanisms remains unknown. In our study, serum hs-CRP levels did not predict the risk of incident knee OA leading to hospitalization.

We found that dietary Mg intake was related to the analyzed risk factors of knee OA, except for age and previous injuries. We also found that those with a lower dietary Mg intake had significantly higher serum hs-CRP concentrations than those with a higher dietary Mg intake. The association was even stronger after adjustment for all covariates than after adjustment for age, gender and energy intake only. This may be partly due to the interference of BMI, which is closely related to serum CRP level [40]. In all, our study substantially adds to previous evidence that low dietary Mg intake contributes to serum hs-CRP levels.

The main strengths of our study were its prospective design and the large nationally representative and well-characterized population sample. The reliability of the diagnoses of knee and hip OA at baseline, and the agreement between clinical and radiological diagnoses proved to be acceptable in the quality control trials carried out as part of field examinations [21, 22, 41]. Furthermore, we had access to information on the major risk factors of OA at baseline. However, although the detailed data on multiple OA risk factors allow adjustment for potential confounders, we cannot rule out the possibility of residual confounding.

The relatively wide confidence intervals of the risk estimates resulting from weak statistical power constitute the most severe limitation of our study. Another limitation of the study is that the follow-up covered knee OA cases admitted to a hospital, but only a part of all patients suffering from knee OA are ever hospitalized. The Finnish public health care system is of high quality and equally available to all Finns. Most knee OA patients are primarily diagnosed in out-patient clinics or occupational health care. Plain radiographs are a standard examination when knee OA is suspected [42] and the American College of Rheumatology clinical/radiographic criteria [43] are also recommended by the Finnish Current Care Guidelines for establishing a knee OA diagnosis [44]. Patients with diagnostic challenges and symptoms severe enough (and concordant with imaging findings) to consider operative treatment are mainly referred to specialists in public hospitals. Those who are hospitalized may not be representative of all people who develop knee OA. Moreover, we were unable to take into consideration the comorbidities of the study participants, which may have affected their eligibility for arthroplasty. We were also unable to distinguish primary from secondary knee OA at follow-up. This was dealt with by taking injuries into account in the data analysis. Our cohort consisted of 70 participants with baseline rheumatoid arthritis and none of these developed incident knee OA. Therefore, it was not possible to control the effect of baseline rheumatoid arthritis on the results using a statistical model. Furthermore, we cannot rule out that some of the participants may have had early knee OA which was missed by the physicians in the baseline survey.

The availability of only questionnaire-based information on dietary Mg intake may present another limitation to our study. The over- and underreporting of foods may affect the calculated dietary Mg intake, and may thus also affect the correlations between the studied variables by causing information bias [45, 46]. Age, gender and obesity have been associated with a higher rate of energy intake underreporting [47–50]. In the Health 2000 survey, dietary Mg intake in FFQ has, however, shown to be sufficiently repeatable and representative of actual food records [26, 27]. Another limitation of the study is the small number of participants with low dietary Mg intake levels, which is probably explained by the high standard of living and good nutrition level in Finland.

In conclusion, the current study suggests that low dietary intake of Mg or elevated serum hs-CRP levels do not predict incident clinical knee OA, although Mg intake is inversely associated with serum hs-CRP. Earlier controversial results may be due to cross-sectional study designs or divergent associations between major OA risk factors and serum hs-CRP levels and dietary Mg intake.
